# ﻿Two new species of *Boesenbergia* (Zingiberaceae), from Sabah, Malaysia

**DOI:** 10.3897/phytokeys.211.83985

**Published:** 2022-10-17

**Authors:** Nyee Fan Lam, Halijah Ibrahim, Yen Yen Sam, Rozainah Mohammad Zakaria, Axel Dalberg Poulsen

**Affiliations:** 1 Faculty of Science, University of Malaya, Jalan Professor Diraja Ungku Aziz, 50603, Kuala Lumpur, Malaysia University of Malaya Kuala Lumpur Malaysia; 2 Institute for Tropical Biology and Conservation, Universiti Malaysia Sabah, Jalan UMS, 88400 Kota Kinabalu, Malaysia Universiti Malaysia Sabah Kota Kinabalu Malaysia; 3 Forest Research Institute Malaysia, Jalan FRIM, 52109 Kepong, Kuala Lumpur, Selangor Malaysia Forest Research Institute Malaysia Kuala Lumpur Malaysia; 4 Royal Botanical Garden, Arboretum PI, Edinburgh, EH3 5NZ, UK Royal Botanical Garden Edinburgh United Kingdom

**Keywords:** *Boesenbergia*, Borneo, hill slope, Sabah

## Abstract

Two new species of *Boesenbergia*, *B.sugudensis***sp. nov.** and *B.truncata***sp. nov.** were discovered in Sabah, Malaysian Borneo. *Boesenbergiasugudensis* resembles *B.imbakensis* in that the leaf sheath of the plant is not thickened and in the anther thecae dehiscing by longitudinal slits, but differs in having a longer petiole and tubular calyx. *Boesenbergiatruncata* resembles *B.orbiculata* by the short petiole and a bilobed calyx, but differs by the truncate leaf base, the acute leaf apex, opposite leaves with a narrower lamina parallel to the ground and anther thecae dehiscing by pores. The new species are described and illustrated in detail.

## ﻿Introduction

*Boesenbergia* Kuntze is one of the genera in the family Zingiberaceae with small-size species. The genus is placed in the subtribe Zingiberae of the tribe Zingiberoideae (Kress et al. 2002) and harbours approximately 99 species distributed in tropical Asia with the centre of diversity in Thailand (28 species) and Borneo (36 species) (Smith 1987; Ibrahim 1992; Sirirugsa 1992; Poulsen 1993; Larsen 1997; Larsen et al. 1999; Cowley 2000; Saensouk and Larsen 2001; Lamb et al. 2013). There were 8 species in Sabah with one variety. The discovery of the two species described below elevates the number of *Boesenbergia* species in Borneo to 38 species.

Smith (1987) pointed out that the important diagnostic characters for Bornean *Boesenbergia* are patterns of anther dehiscence, characteristics of seeds, variegation of leaf and character of leaf-shoots. Sakai and Nagamasu (2006, 2009) added arrangement of flowers in inflorescence as another character. Meanwhile, different characters were used for the diagnosis of *Boesenbergialongiflora* complex from Indochina, namely, flowers per inflorescences, flower colour, labellum pattern colour, shape and measurement, floral tube length, androecial tube length, anther length and underground architecture (Mood et al. 2013).

## ﻿Materials and methods

The morphology of the two new species was analyzed using herbarium materials (AAU, C, E, K, KEP, KUL, L, P, SAN, SING, and SNP) and living plants. During fieldwork, careful observations and measurements of morphological characters were recorded prior to preparation of herbarium specimens in the field. When possible, up to four duplicates of each collection were made, and a sample of a fresh leaf was preserved in silica gel for genetic studies. Voucher specimens were deposited at BORH and SAN. Floral bracts and flowers were immediately fixed in FAA (formaldehyde: glacial acetic acid: alcohol).

Field observations included habit; rhizome (diameter, shape, colour, indumentum); leafy shoot (height and distance between adjacent shoots); leaf sheath (number, length, colour, indumentum); ligule (length, shape of apex, colour, indumentum); petiole (length, shape (whether channelled or rounded in cross section), colour, indumentum); number of leaves per leafy-shoot; lamina (size, shape, aspect of lamina (whether held at a certain angle), venation, texture, colour (on both surfaces), indumentum, base, margin, apex); inflorescence size, floral bracts arrangement, and size, shape, texture, colour, indumentum, bracteoles (colour, hairiness, texture, shape); calyx, corolla, labellum, staminode, stamen, ovary, fruit, seed and aril.

### ﻿Data resources

The data underpinning the analysis reported in this paper are deposited at GBIF, the Global Biodiversity Information Facility, and are available at https://doi.org/10.15468/4c4gag.

### ﻿Key to species of Bornean *Boesenbergia* (modified from Sakai and Nagamasu 2009)

**Table:** 

1	Creeping herbs; shoots normally single-leaved; inflorescence more or less sessile; anther dehiscing by slits	**2**
–	Erect herbs; shoots with one, to many leaves; inflorescence sessile or long pedunculate; anther dehiscing by slits or pores	**6**
2	Leaves more or less circular, obtuse or obscurely emarginate at apex	***B.orbiculata* R.M. Sm**
–	Leaves elliptic or lanceolate, acute at apex	**3**
3	Leaves plain green	**4**
–	Leaves variegated	**5**
4	Corolla tube pubescent outside; flowers not red at throat; labellum entire	***B.flavoalba* R.M. Sm**
–	Corolla tube glabrous outside; flowers red at throat labellum bilobed	***B.flavorubra* R.M. Sm**
5	Petioles 2–3 cm; lamina 7–12 by 2.5–7 cm, dark green with a band of lighter green up the midrib, variegation sometimes extending to the main lateral veins	***B.variegata* R.M. Sm**
–	Petiole to 0.5 cm, lamina 4–8 by 1.5–2 cm, mid green with a broad silver band on either side of the midrib above	***B.kerbyi* R.M. Sm**
6	Fertile shoots single-leaved, rarely bladeless or 2– or 3-leaved	**7**
–	Fertile shoots with two or more leaves	**11**
7	Lamina 50 by 12 cm or larger	***B.grandifolia* (Valeton) Merr**
–	Lamina much smaller, not exceeding 30 cm long	**8**
8	Lamina deeply cordate at the base	***B.cordata* R.M. Sm**
–	Base of the lamina more or less attenutate not cordate	**9**
9	Petiole 17–34 cm long	***B.bruneiana* Cowley**
–	Petiole not exceeding 17 cm	**10**
10	Lamina 7–12 cm wide; petiole robust ca. 5 mm thick; lamina with appressed hairs especially around midrib below	***B.lambirensis* S. Sakai & Nagam**
–	Lamina less than 7 cm wide; petiole slender, 2 mm or less thick; leaves glabrous	***B.ischonosiphon* S. Sakai & Nagam**
11	Outermost bract forming a bucket or vase-like structure enclosing inflorescence sometimes together with sheaths of upper leaves; lamina large, much longer than 30 cm	**12**
–	Leaf base or sheaths not thickened as above, or if thickened, leaves are much shorter	**14**
12	Petiole 42–50 cm long	***B.jangarunii* Cowley**
–	Leaf base long-attenuate forming a winged petiole less than 25 cm long	**13**
13	Inflorescence densely pubescent; anther ca. 3 mm long, dehiscing by subapical pores	***B.hosensis* Cowley**
–	Plant almost glabrous, anther ca. 10 mm long, dehiscing for ca. 6 mm long (probably dehiscing by slits)	***B.armeniaca* Cowley**
14	Leaves larger than 20 by 7 cm; anthers dehiscing by pores	**15**
–	Leaves shorter than 20 cm, if longer narrower than 7 cm; anthers dehiscing by slits or pores	**16**
15	Leaf sheath sparsely hairy or glabrous; bracts 5–8 cm; corolla tube 8–10 cm; ovary glabrous	***B.grandis* R.M. Sm**
–	Leaf sheath densely hairy; bracts 2–3.5 cm long; corolla tube ca. 5.5 cm long; ovary densely hairy in upper half	***B.lysichitoides* S. Sakai & Nagam**
16	Inflorescence long-exserted from the leaf sheaths when fully grown, spindle-shaped; flowers red and white	***B.pulchella* (Ridl.) Merr**
–	Inflorescence never long-exserted or spindle-shaped; flower colours variable	**17**
17	Leaves linear, arrangement of blades strongly flabellate	**18**
–	Leaves elliptic, lanceolate or rarely linear-lanceolate, arrangement of blades never flabellate	**19**
18	Flowers plain yellow; anther dehiscing by apical pores; bracts 3.5–6.5 cm	***B.flabellata* S. Sakai & Nagam**
–	Flowers white, yellow in the centre, pink at the base; anther dehiscing by slits; bracts up to 3 cm	***B.burttiana* R.M. Sm**
19	Leaves variegated	**20**
–	Leaves plain green	**24**
20	Leaves bullate, dark green around main veins and almost silvery on raised areas	***B.hutchinsoniana* B.L. Burtt & R.M. Sm**
–	Leaves smooth with a silverish or light green central cloud	**21**
21	Petiole never exceeding 3 cm, lamina oblanceolate with attenuate base	***B.hirta* (Ridl.) Merr**
–	Petiole usually much longer than 3 cm, lamina lanceolate to elliptic with cuneate base	**22**
22	Leaves with a silver cloud; flowers yellow, labellum orange spotted	***B.ornata* (N.E. Br.) R.M. Sm**
–	Leaves with yellow cloud, flowers orange or white with some yellow and reddish purple	**23**
23	Leaves 5–12 by 3–4 cm; flowers orange, darker at base of labellum; anther dehiscing throughout its length	***B.aurantiaca* R.M. Sm**
–	Leaves 18–23 by 4–6 cm; flowers white with some yellow and reddish purple; anther dehiscing by apical pores, or anther dehiscent only in upper 2/3	***B.belalongensis* A.D. Poulsen**
24	At least a few uppermost leaf sheaths thickened and forming a cup-shaped structure	**25**
–	Leaf sheath not thickened as above	**26**
25	Innermost leaf sheaths enclosing inflorescence much shorter and wider than outer ones; leaves drying darkish brown	***B.laevivaginata* S. Sakai & Nagam**
–	All leaves with more or less equal laminae; leaves green or grey-green when dry	***B.urceoligena* A. D. Poulsen**
26	Anther dehiscing by slits throughout their length	**27**
–	Anther dehiscing by pores	**28**
27	Petiole less than 8 cm; bracts ca. 2.6 × 0.4 cm; calyx 3-lobed	***B.imbakensis* S. Sakai & Nagam**
–	Petiole more than 10 cm, Calyx tubular	***B.sugudensis* N.F. Lam, sp. nov.**
28	Lamina wider than 4 cm, petiole 2 cm, lamina 5.2–6.5 × 3.4–3.6 cm	***B.truncata* N.F. Lam, sp. nov.**
–	Width of lamina less than 4 cm	**29**
29	Lamina narrowly lanceolate, lamina 12–20 by 1.5–3 cm; petiole usually 7–8 cm	***B.stenophylla* R.M. Sm**
–	Lamina much shorter, up to 12 cm long, if longer, petiole much shorter than 7 cm	**30**
30	Leaf sheath and ligule long pubescent	***B.parva* (Ridl.) Merr**
–	Leaf sheath and ligule almost glabrous	**31**
31	Flowers yellow-orange	***B.oligosperma* (K. Schum.) R.M. Sm**
–	Flowers white and yellow occasionally with red in throat	***B.subulata* S. Sakai & Nagam**

## ﻿Taxonomy

### ﻿ Boesenbergia sugudensis

Taxon classificationPlantaeZingiberalesZingiberaceae

N.F.Lam sp. nov.

4BDB2AC7-935E-562F-AD06-68890EB5D4B9

urn:lsid:ipni.org:names:77306651-1

Figs 1 , 2

#### Diagnosis.

The new species resembles *B.imbakensis* S. Sakai & Nagam. in that the leaf sheaths are not thickened and in the anther thecae dehiscing longitudinally, but differs in having a longer petiole (>10 cm vs. 4–7.5 cm) and bilobed apex of calyx (vs. trilobed) (Table 1).

**Table 1.** Distinguishing morphological characters of *Boesenbergiasugudensis*, *B.imbakensis*, *B.truncata* and *B.orbiculata*.

Characters	Species			
	*B.sugudensis*	*B.imbakensis*	*B.truncata*	*B.orbiculata*
Plant height (cm)	44	30	11.5	8
Rhizome	Fibrous	Small	Fibrous, sections of rhizome with 1–2 cm long and papery texture bracts	Unknown
Ligule	0.4 cm, caudate, brownish green, glabrous	1 cm, triangular, green, glabrous	0.3 cm, entire, light brown, glabrous	2 cm, bilobed, lobes rounded, glabrous
Petiole	22.5 cm, green, base reddish up to middle part	4–7.5 cm, green, base reddish up to middle	2 cm long, green, base reddish up to middle part	2 cm
Lamina	Elliptic, upper surface dark green, lower surface paler	Narrowly ovate to obovate, plain green	Elliptic, unequal/oblique?, upper surface dark green, lower surface lighter green	sub-orbiculate, upper surface pale green, lower surface light green
Leaf size (cm)	21 × 7.3	11–16 × 3–4	5.2–6.5 × 3.4–3.6	5–8 × 4–7
Leaf base	Rounded	Attenuate	Truncated to sub-cordate	Sub-cordate
Leaf apex	Acuminate, acumen ca. 3 mm	Slightly acuminate, acumen ca. 1-2 mm	Acute, acumen ca. 1 mm	Sub-obicular, obtuse or occasionally shallowly emarginate
Bracts	6.5 cm, translucent white, linear elliptic, glabrous	2.6 × 0.4 cm, narrowly ovate, membranous	1.8 × 3 cm, white, narrowly ovate, pubescent	2.5 cm, boat-shaped, whitish brown, sparsely pubescent,
Calyx	Tubular, apex bilobed, glabrous	Tubular, apex unequally and shallowly 3-lobed, glabrous	Tubular, apex bilobed, pubescent	Unilaterally split, apex bilobed
Labellum	White with narrow light red band from base until the middle, yellow spread towards the lip, 1.3 × 1 cm	White with yellow on the centre and red at the throat, 1.8 × 1.4 cm	White with yellow band at base in the middle, spread towards lip, 0.6 × 0.5 cm	White with deep yellow in the centre and a red mark at the base, 1 × 1 cm
Lateral corolla lobe	Glabrous, white, 1.7 × 0.3 cm, ovate, apex rounded, longer than labellum	Glabrous, white, 1.4 × 0.4 cm	Pubescent, white, 0.3 × 0.1 cm	1 cm long
Anther	Upper and lower surfaces pubescent, 0.6 cm	Glabrous on ventral, shortly pubescent on the dorsal surface, 0.6 cm	Glabrous, 0.4 cm long	Slightly pubescent, 0.4–0.5 cm
Anther dehiscent	Slit	Slit	Pore	Slit
Stigma	Cup-shaped, white, glabrous	Unknown	Emarginate, white, glabrous	Shape and colour unknown, glabrous

**Figure 1.**
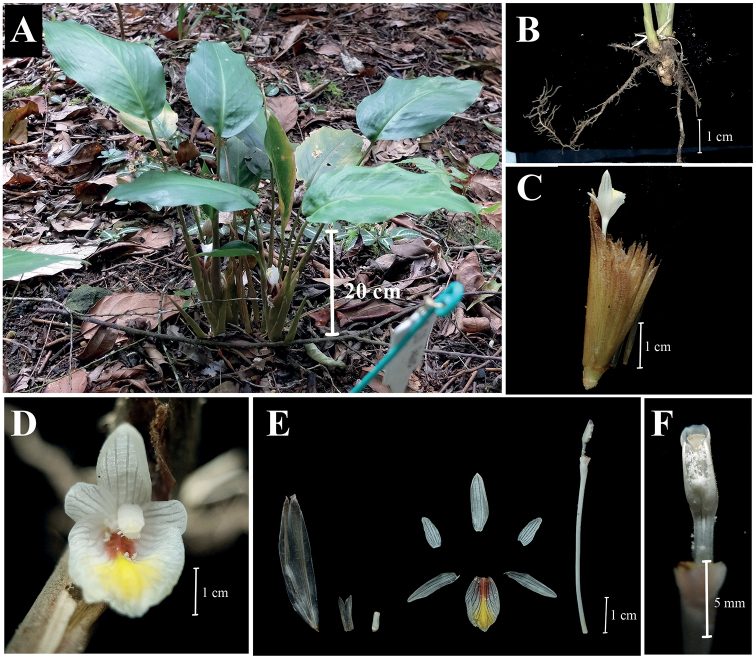
*Boesenbergiasugudensis***A** habit **B** rhizome and roots **C** spike with one open flower **D** flower **E** bracteole, calyx, corolla lobes, staminodes, labellum, floral tube with stamen **F** stamen, ventral view (Photographed by Lam Nyee Fan).

#### Type.

Malaysia. Borneo. Sabah. cult. at Kipandi Park, Moyog, 05°54.68'N, 116°06.27'E, 700 m elevation, 12 October 2016, *Lam Nyee Fan 356* (holotype BORH!, isotype SAN). Original material collected by Linus Gokusing (BS-23) at Kampung (Kg.) Sugud, Penampang, Sabah, 05°50.23'N, 116°06.60'E, 50–100 m elevation.

**Figure 2.**
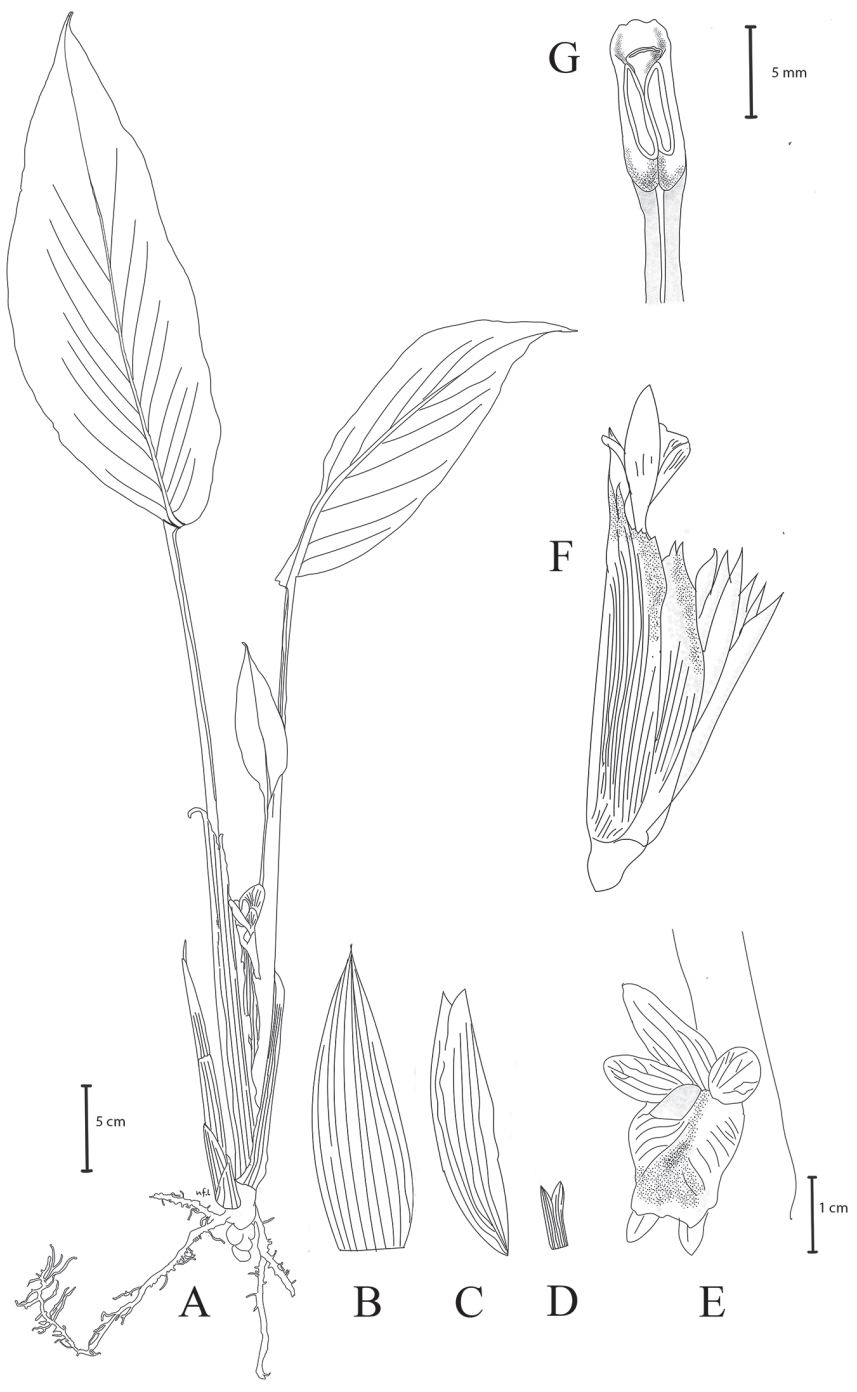
*Boesenbergiasugudensis* Lam N.F., sp. nov. **A** habit **B** bract **C** bracteole **D** calyx **E** flower **F** spike with one open flower **G** stamen, ventral view (Drawing by Lam Nyee Fan). Scale bars: 5 cm (**A**); 1 cm (**B, C, D, E, F**); 5 mm (**G**).

#### Description.

Terrestrial, evergreen, herb. *Rhizome* fibrous, subterranean, ca. 0.8 cm in diameter, base ca. 1.5 cm in diameter, roots white. *Leafy shoots* 44 cm tall, with 2–3 leaves forming a loose pseudostem, erect, ca. 13 cm long, with 2–3 outer leafless sheaths, 3.9–12.5 × 0.8–1.25 cm, green, pubescent on outer surface and glabrous on inner surface, veins 1 mm apart. *Ligule* ca. 0.4 cm long, caudate, brownish green, glabrous. *Petiole* 12–22.5 cm long, canaliculate, green, reddish in lower half. *Lamina* elliptic, 20–22 × 6.5–7.5 cm, erect, dark green above, pale green beneath, glabrous above, pubescent beneath, base rounded, margin entire, glabrous, apex acuminate, with acumen ca. 3 mm. *Inflorescence* ca. 4.7 cm, peduncle 0.8 cm, spike ca. 7.2 × 3 cm. flowers arranged in one-sided spiral, 18 flowers including 5 new buds and 4 old buds, one flower open at a time. *Fertile bracts* linear elliptic, ca. 6.5 cm long, translucent pubescent on outer surface and glabrous on inner surface, margin entire, apex attenuate. *Bracteoles* elliptic, ca. 3.8 × 0.8 cm, translucent, pubescent on outer surface and glabrous on inner surface, margin entire, apex acute. *Flower* white, born singly from each bract and bracteole; calyx 1 cm long, tubular, 2-lobed, translucent, pubescent on both surfaces; corolla tube ca. 4.6 cm long, ca. 1.2 mm wide at base, lobes white, glabrous throughout, dorsal lobe ovate-oblong, ca. 1.7 × 0.45 cm, concave, erect, apex acute, lateral lobes ovate, ca. 1.7 × 0.3 cm, clasping the labellum and extending 4 mm beyond, apex rounded; labellum, obovate-elliptic, ca. 1.3 cm × 1 cm curved-backward, with a narrow light red band in the centre lower half, yellow towards apex, glabrous; lateral staminodes white, narrowly obovate, ca. 1.5 × 0.5 cm, glabrous; stamen white throughout, ca. 5.7 cm long, filament ca. 3.5 × 1.2 mm (widest at base), pubescent adaxially and abaxially, anther ca. 0.3 × 0.2 cm, glabrous, anther crest ca. 2 × 3 mm, bilobed, glabrous, thecae oblong, ca. 0.2 × 0.1 cm, glabrous, dehiscing longitudinally for its entire length; ovary ca. 5 × 2 mm, 8.6 cm, stigma cup-shaped, glabrous; epigynous glands two, ca. 0.45 cm long, linear, apex truncate, white. *Fruit* not seen.

#### Distribution.

Endemic in Borneo, Sabah; known only from the type locality, Kg. Sugud.

#### Etymology.

The species epithet refers to the location where the type was collected.

#### Ecology.

Primary forest in lowlands, hill slope at 50–100 m elevation.

#### Conservation status.

Data Deficient (DD). The taxon was assessed using the criteria described in IUCN (2001). The taxon is endemic to Sabah and only found at Kg. Sugud, Penampang, Sabah, Malaysia. One population was observed at the site where specimens were collected.

### ﻿ Boesenbergia truncata

Taxon classificationPlantaeZingiberalesZingiberaceae

N.F.Lam sp. nov.

9D98C117-C58A-59AE-BA08-988DCD61959D

urn:lsid:ipni.org:names:77306652-1

Figs 3 , 4 , 5

#### Diagnosis.

The new species resembles *B.orbiculata* by the short petiole (c. 2 cm long) and the bilobed calyx, but differs in having truncate leaf base, an acute leaf apex (vs. sub-obicular, obtuse, or occasionally retuse), paired opposite leaves, and lamina parallel to the ground (vs. a single shoot), anther thecae dehiscing by pore (vs. slit), and the lamina slightly narrow (3.4–3.6 cm vs. 4–7 cm) (Table 1).

**Figure 3.**
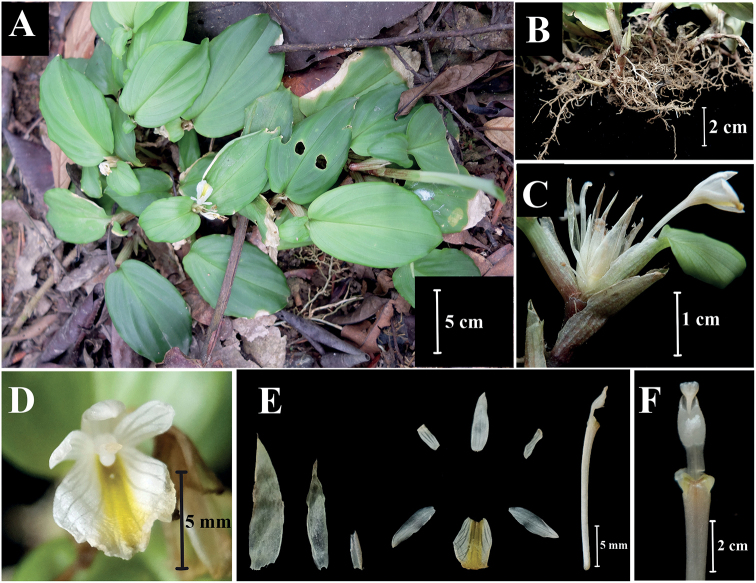
*Boesenbergiatruncata***A** habit **B** rhizome and roots **C** spike with one open flower **D** flower **E** ract, bracteole, calyx, corolla lobes, staminodes, labellum, floral tube with stamen **F** stamen, ventral view (Photographed by Lam Nyee Fan).

#### Type.

Malaysia. Borneo. Sabah. cult. at Kipandi Park, Moyog, 05°54.68'N, 116°06.27'E, 700 m elevation. 12 October 2016, *Lam Nyee Fan 342* (holotype BORH!, isotype SAN). Original material collected near the park, by Linus Gokusing (BS-09), 100 m west of Kipandi Park, Sabah, 05°52.28'N, 116°14.95'E, 700 m elevation.

**Figure 4.**
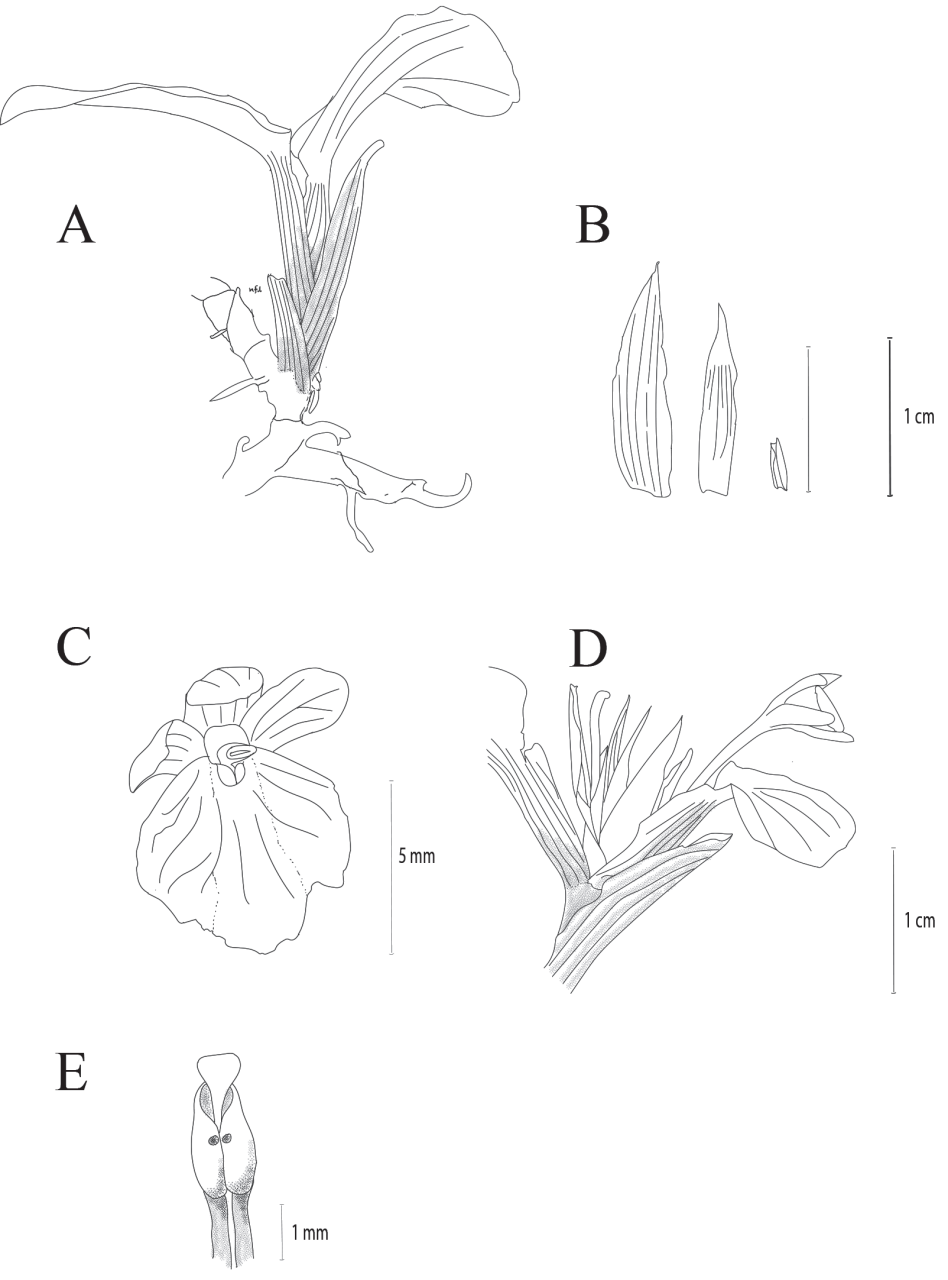
*Boesenbergiatruncata* Lam N.F., sp. nov. **A** habit, lateral view **B** bract **C** bracteole **D** calyx **E** flower **F** spike with one open flower **G** stamen, ventral view (Drawing by Lam Nyee Fan). Scale bars: 1 cm (**A, B**); 5 mm (**C**); 1 cm (**D**); 1 mm (**E**).

#### Description.

Terrestrial, evergreen, herb. *Rhizome* fibrous, subterranean, ca. 2 cm long internodes, base ca. 0.4 cm in diameter, light brown, roots white, ca. 5 cm long. *Leafy shoots* ca. 11.5 cm tall, with erect pseudostem ca. 1.5 cm long, with 1 sheath, ca. 1.4 × 0.6 cm, glabrous, veins 1 mm apart, green with light purple at base, margins entire. *Ligule* 0.3 cm long, entire, light brown, glabrous. *Petiole* 1.4–2 cm long, canaliculate, green, base reddish up to middle. Leafy shoots with two leaves opposite to each other and parallel to the ground. *Lamina* unequal elliptic, 5.2–6.5 × 3.4–3.6 cm, dark green above, lighter green beneath, glabrous, margin entire; base truncated, apex acute with acumen ca. 1 mm. *Inflorescence* ca. 2.5 × 1.5 cm, peduncle ca. 0.45 cm, flowers arranged in one-sided spiral, 8 flowers including one new bud and 1 old bud, one flower open at a time. *Fertile bracts* narrowly lanceolate, ca. 1.8 × 3 cm, white, outer and inner surfaces pubescent, margin entire, apex caudate. *Bracteoles* linear elliptic, ca. 1.5 × 2 cm, white, outer and inner surfaces pubescent, margin entire, apex acuminate. *Flower* white, born singly from each bract and bracteole, calyx 0.4 cm long, tubular, white, pubescent, corolla tube-white, pubescent, apex acute, dorsal lobe lanceolate, ca. 0.8 × 0.2 cm, concave, lateral lobes elliptic, ca. 0.6 × 0.2 cm, labellum obovate, ca. 0.6 cm × 0.5 cm, yellow band at base in the centre until the apex, curved-backward, lateral staminodes oblong, ca. 0.3 × 0.1 cm, white, tip rounded, pubescent, stamen ca. 2.3 cm long; filament ca. 4 × 1 mm (widest at base), glabrous adaxially and abaxially, anther ca. 2 mm long, glabrous; anther crest bilobed, glabrous; thecae oblong, ca. 0.2 × 0.1 cm, white, pubescent, dehiscing by pore, stigma emarginate, white, glabrous. Fruit not seen.

**Figure 5.**
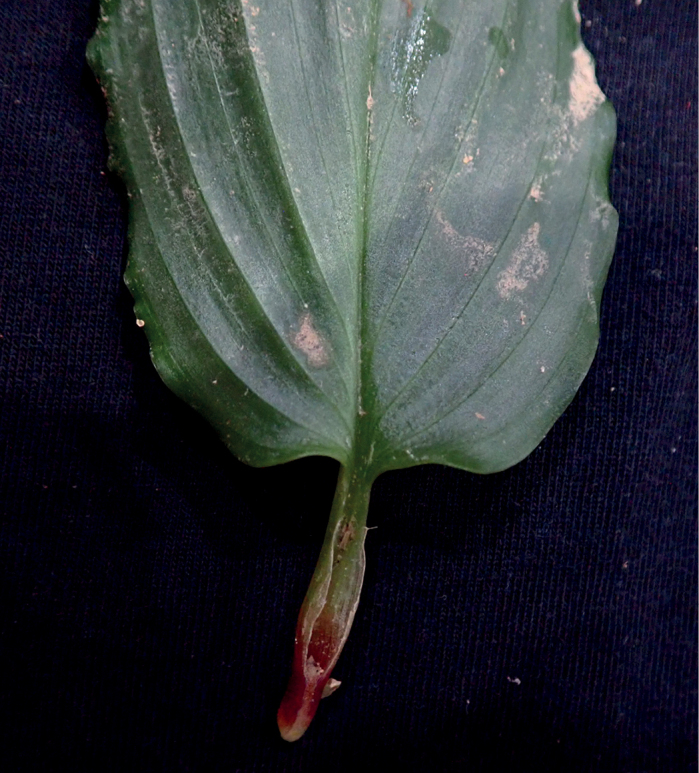
Shape of base of leaf of *Boesenbergiatruncata*.

#### Distribution.

Endemic in Borneo, Sabah; known only from Kipandi Park of Crocker Range.

#### Etymology.

The species epithet refers to truncated leaf base.

#### Ecology.

Primary forest, hill slope at 500–800 m elevation.

#### Conservation status.

Vulnerable (VU D2). The taxon was assessed using criteria described in IUCN (2001). The taxon is endemic to Sabah and only found at Crocker Range, Sabah, Malaysia. There were only 3 populations found at the site of collection. This taxon is not found outside the type locality.

## Supplementary Material

XML Treatment for Boesenbergia sugudensis

XML Treatment for Boesenbergia truncata
